# Supercritical Carbon Dioxide Extraction of the Oak Silkworm (*Antheraea pernyi*) Pupal Oil: Process Optimization and Composition Determination

**DOI:** 10.3390/ijms13022354

**Published:** 2012-02-21

**Authors:** Wen-Juan Pan, Ai-Mei Liao, Jian-Guo Zhang, Zeng Dong, Zhao-Jun Wei

**Affiliations:** 1 School of Biotechnology and Food Engineering, Hefei University of Technology, Hefei 230009, China; E-Mails: wenjuanpan12345@163.com (W.-J.P.); liaoaimei100@163.com (A.-M.L.); zjghfut@sina.com (J.-G.Z.); dongozeng@163.com (Z.D.); 2 Department of Life Science, Hefei Normal University, Hefei 230061, China

**Keywords:** supercritical carbon dioxide, oak silkworm pupal oil, response surface methodology

## Abstract

Supercritical carbon dioxide (SC-CO_2_) extraction of oil from oak silkworm pupae was performed in the present research. Response surface methodology (RSM) was applied to optimize the parameters of SC-CO_2_ extraction, including extraction pressure, temperature, time and CO_2_ flow rate on the yield of oak silkworm pupal oil (OSPO). The optimal extraction condition for oil yield within the experimental range of the variables researched was at 28.03 MPa, 1.83 h, 35.31 °C and 20.26 L/h as flow rate of CO_2_. Under this condition, the oil yield was predicted to be 26.18%. The oak silkworm pupal oil contains eight fatty acids, and is rich in unsaturated fatty acids and α-linolenic acid (ALA), accounting for 77.29% and 34.27% in the total oil respectively.

## 1. Introduction

Three species of silkworms, the mulberry silkworm (*Bombyx mori* L.), the oak silkworm (*Antheraea pernyi*) and the eri silkworm (*Samia cynthia ricini*), are widely reared around the world for the production of silk thread. The former two silkworms come from China, with the third originating in from India [1]. Silkworm pupae are known for their nutritional value, due to the presence of high protein and high fat (about 30% of the total dry pupae weight). The protein in silkworm pupae contains 18 known amino acids, which include all of the essential amino acids and sulfur-containing amino acids, exhibiting high quality, according to the amino acid profile recommended by the Food and Agriculture Organization (FAO)/World Health Organization (WHO) [2–4]. The mulberry silkworm *B. mori* reared on mulberry leaves belongs to family Bombycidae, while the non-mulberry silkworm *A. pernyi* and *S. cynthia ricini* reared on oak and castor leaves respectively, belong to the Saturniid family [5]. Due to the different food resources, the proximate compositions (%) for non-mulberry and mulberry silkworm pupae were different to each other in many ways: total protein (12–16%), total fat (11–20%) and carbohydrate (1.2–1.8%) [5]. The mulberry silkworm pupal oil extracted from the de-silked pupae contained more than 68% total unsaturated fatty acids and 27.99% alpha-linolenic acid (ALA) [6]. Although the large-scale mulberry silkworm pupae were usually obtained from the de-silked cocoon, the high temperature of the boiling water combined with alkali during the silk-drawing process may affect the nutritional value of the silkworm pupal oil. Even if supercritical carbon dioxide (SC-CO_2_) used for extraction, long-term exposure to alkali is still harmful to human health. In China, it is very common for people to rear the oak silkworm for eating and silkworm pupae have also been approved as a new food resource in Zhou and Han, 2006 [7]. However knowledge about the nutritional composition of oak silkworm *A. pernyi* pupae is still rare.

Recently, supercritical carbon dioxide (SC-CO2) extraction has become wildly used in different fields, such as food science, natural products, bioactive compounds, by-product recovery and the pharmaceutical and environmental sciences [8,9]. Separation of β-sitosterol, oils or some special fatty acids (e.g., gamma-linolenic, polyunsaturated fatty acid) also can be performed by SC-CO_2_ extraction [10]. Compared with conventional technology, supercritical carbon dioxide (SC-CO_2_) extraction has many advantages, including: non-explosive, nontoxic and non-solvent residues, high purity and low cost [10–12]. SC-CO_2_ systems can be operated at low temperatures which prevent degradation associated with heat induction [13]. In addition, CO_2_ can be easily removed from the extract when pressure and temperature are reduced below its critical condition. The efficiency of supercritical extraction, as well as subcritical extraction, can be affected by several variables, which include temperature, pressure, extraction time and carbon dioxide flow rate [6]. Response surface methodology (RSM) is an effective and powerful statistical method for optimizing experimental conditions and the investigation of critical processes with a reduced number of experimental trials [6]. Over the past several years, RSM has been successfully employed to optimize the supercritical CO_2_ extraction of cottonseed oil [14], soybean oil [15], pomegranate seed oil [16], cottonseed oil [14], rosehip seed oil [17], pumpkin seed oil [18] and tea seed Oil [19]. Here, we report our work on oil extraction from oak silkworm pupae with SC-CO_2_ in optimized conditions which was found with the help of robust RSM.

## 2. Results and Discussion

### 2.1. Fitting the Model

The experimental design was adopted on the basis of coded levels from four independent variables (Table 1) to minimize the number of experimental runs and the time needed for optimizing oil extraction conditions from oak silkworm pupae, resulting in a 31-set simplified experimental set (Table 2). To obtain a regression equation that could predict the response within the given range, independent and dependent variables were analyzed. To evaluate the significance of each coefficient and indicate the interaction strength of each parameter, the ANOVA (*F*-test) and *p*-values are used (Table 3). The model with a *p*-value less than 0.001 was statistically significant, which implied the model was suitable for this experiment. Meanwhile, the “lack of fit” of this model with the *p*-value of 0.21 was insignificant, indicating that the accuracy and general availability of the polynomial model are adequate. The coefficient of determination (*R*^2^) and adjusted coefficient of determination (*Adj. R*^2^), with values of 0.9178 and 0.8459, are also shown in Table 3. Regression coefficients of intercept, linear, quadratic, and interaction terms of the model which are presented in Table 4 were calculated by using a least squares technique.

To calculate the coefficients of the second-order polynomial equation and the obtained regression coefficients, multivariable linear regression was used and its significance determined by the Student *t* test and *p*-value (Table 4). More significance will be concluded with a larger absolute *F*-value and a smaller *p*-value [20]. Neglecting the non-significant parameters, the final predictive equation obtained is showed as below (Equation (1)):

(1) $$\begin{array}{l} \text{Y}=25.9143+0.4521{\text{X}}_{1}+0.1621{\text{X}}_{2}+0.4196{\text{X}}_{3}+0.1346{\text{X}}_{4}-0.4389{\text{X}}_{1}{\text{X}}_{1}-\\ 0.3256{\text{X}}_{1}{\text{X}}_{2}-0.2056{\text{X}}_{1}{\text{X}}_{4}-0.5564\;{\text{X}}_{2}{\text{X}}_{2}-0.4056{\text{X}}_{2}{\text{X}}_{4}-0.3814{\text{X}}_{3}{\text{X}}_{3}-0.1551{\text{X}}_{4}{\text{X}}_{4} \end{array}$$

Table 4 and Equation (1) showed that the factors most significantly affecting oil yield was the two linear term of pressure and time (*p* < 0.001), followed by the three quadratic term of temperature, pressure and time (*p* < 0.001). The interactions between pressure and temperature, temperature and SC-CO_2_ flow rate also had highly significant effects on oil yield. Based on the above model, the optimal condition for oak silkworm pupal oil yield was: 28.03 MPa, 35.31 °C, 1.83 h, and 20.26 L/h, and the oil yield was 26.18% under this condition.

### 2.2. Analysis of Response Surface

According to Equation (1), three-dimensional response surface curves and contour plots were plotted to determine their optimum values and to analyze the interactions among the various selected factors for obtaining the maximum oil recovery. The plots were generated by plotting the response using the *z*-axis against two independent variables while keeping the other two independent variables at their zero level.

**Figure 1:**
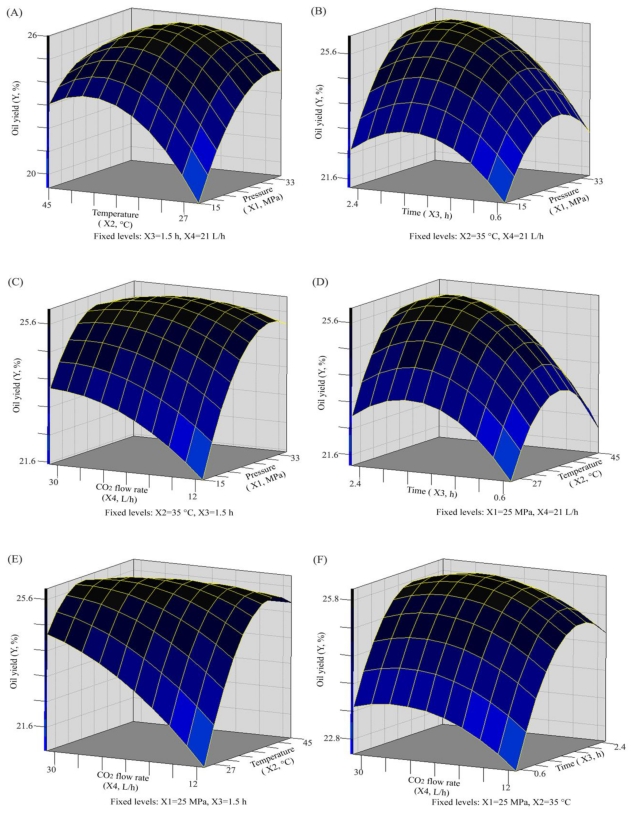
Response surface plots showing the effects of different factors on oil yield. X_1_: Pressure (MPa); X_2_: Temperature (°C); X_3_: Extraction time (h); X_4_: CO_2_ flow rate (L/h); Y: Oil yield (%).

The best way to visualize the influence of the independent variables on the dependent one is to draw surface response plots of the model [21,22]. The generated response surfaces developed using the fitted quadratic polynomial equation obtained from regression analysis are shown in Figure 1.

Because the solubility of lipids depends largely on the balance between fluid density and solute vapor pressure, which were controlled by pressure and temperature, extraction pressure and temperature are the main parameters that influence extraction efficiency. Figure 1A shows the interaction between extraction pressure and extraction temperature on oil recovery from oak silkworm pupae, while the extraction time and CO_2_ flow rate are respectively fixed at 1.5 h and 21 L/h. At a given extraction temperature, the yield of oil significantly increased with increasing pressure and then decreased with increasing pressure after the temperature reached the center point. This trend became more obvious at lower temperatures. Similar phenomena were also reported for the extraction of *Passiflora* seed oil [23,24] and yellow horn seed oil [25] by SF-CO_2_. This influence may be due to the fact that an elevated extraction pressure at a given temperature will result in an increase in fluid density, which means an enhanced solubility of the oil [6,21]. The solubility of vegetable oils extracted by SC-CO_2_ also varies considerably with temperature and pressure. The oil increases as the pressure increases, basically in the range of 345–550 bar. Solubility increases with an increase in temperature, when the pressure is higher than 345 bar. Conversely, this effect does not occur with pressures lower than 345 bar [26]. This behavior is related to the density of SC-CO_2_. However, high pressure is not always recommended since the increased repulsing solute-solvent interactions from the highly compressed CO_2_ at high-pressure levels will potentially induce complex extraction and difficult analysis [27,28].

Figure 1B describes the effect of extraction pressure and time on oil yield. The results indicated that the oil yield increased gradually with the increase of extraction time at a lower fixed extraction pressure, while the oil yield decreased gradually with the increase of extraction time at the higher extraction pressure. When the extraction pressure lay in the center point up and down, the oil yield increased rapidly with increasing extraction time. At this point oil yield decreased linearly with increased extraction time.

The interaction between extraction pressure and CO_2_ flow rate shown in Figure 1C reveals that the oak silkworm pupal oil yield increases slowly with an increase in CO_2_ flow rate at a lower fixed extraction pressure; then, a decrease of CO_2_ flow rate after the center point of pressure. Before the center extraction pressure, the CO_2_ flow rate only led to a gradual increase in oil yield, especially beyond 21 MPa, when no obvious effect was observed.

Figure 1D illustrates the interaction between extraction time and extraction temperature on oil yield. It was observed that at a given temperature, especially at low or high temperatures, oil yield changed dramatically with extraction time. At the temperature center point, the oil yield rapidly increased with the extraction time.

Figure 1E shows the response surface and contour plots of the effect of extraction temperature and CO_2_ flow rate on the oil yield, with a fixed extraction pressure and extraction time at 25 MPa and 1.5 h, respectively. The CO_2_ flow rate displayed a positive effect on the oil yield at low temperature. However, no obvious effect of CO_2_ flow rate on the oak silkworm pupal oil was observed at a high extraction temperature.

The response surface for the oil yield as related to time and CO_2_ flow rate with a fixed extraction pressure of 25 MPa and temperature of 35 °C is shown as a three-dimensional plot in Figure 1F. It can be seen that no significant effect on oil yield was observed when the extraction time was fixed and the CO_2_ flow rate increased. However, the obvious decreasing trends in oil yield with the extraction time were displayed when the CO_2_ flow rate was fixed. Under a given pressure, temperature or CO_2_ flow rate (Figure 1B,D and F), oil yields decreased slightly after 2.1 h extraction. The above phenomena were difficult to explain, however similar phenomena were also reported for the extraction of other oils by SF-CO_2_, e.g., *Passiflora* seed oil [23,24], yellow horn seed oil [25] and almond oil [29].

### 2.3. Determination of Fatty Acid Composition of Extracted Oils

**Figure 2:**
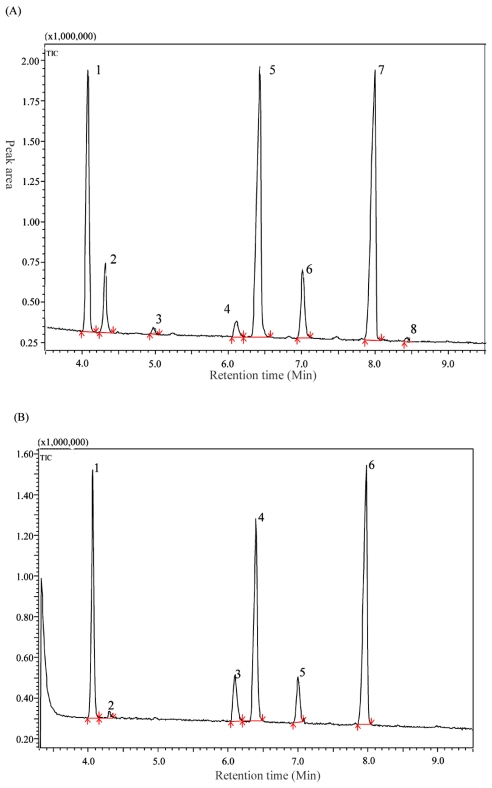
GC chromatogram of FAME of two kind of silkworm pupal oil. A: the Oak silkworm pupal oil; B: the mulberry silkworm pupal oil.

The fatty acid components of the oak and mulberry silkworm pupal oil were analyzed by Gas chromatography-mass spectroscopy (GC/MS). The total ion chromatograms of silkworm pupal oil extracted by supercritical CO_2_ are shown in Figure 2. Eight compounds were identified from the oak silkworm pupal oil, including palmitic acid, palmitoleic acid, heptadecanoic acid, stearic acid, oleic acid, linoleic acid, α-linolenic acid and 10(*z*),13(*z*),16(*z*)-nonadecatrienoic acid. Palmitic acid, α-linolenic acid and oleic acid, as the main components in oak silkworm pupal oil showed 19.92%, 34.27% and 30.97% respectively in the area of the peaks from GC. The heptadecanoic acid and 10(*z*),13(*z*),16(*z*)-nonadecatrienoic acid, however, were not detected in mulberry silkworm pupal oil (Table 5). The oak silkworm pupal oil is rich in unsaturated fatty acids (77.29% of the total fatty acids) including monounsaturated fatty acids in 35.74% and polyunsaturated fatty acids in 41.55%. All of these values are higher than the value for the corresponding unsaturated fatty acid in the mulberry silkworm pupal oil (the corresponding proportions are 64.64%, 26.61% and 38.03%, respectively).

Oak silkworm pupal oil contains a slightly lower proportion of α-linoleic acid (34.27%) than the oils from mulberry silkworm pupae (38.02%), but higher than those in soybeans and sunflowers [15]. In our previous results, α-linoleic acid in spent mulberry silkworm pupae is 27.99% [6], which is lower than that extracted from fresh mulberry silkworm pupae (38.02%) in the present research. The above results demonstrate that the conditions of drawing silk from cocoons (e.g., high temperature, alkali, boiling water) may affect the composition of the silkworm pupal oil. Shanker *et al*. [30] also demonstrated that the fat content and its composition are influenced by species, season, geographical regions, age, and process methods. α-linolenic acid in the diet can prevent disorders such as atherosclerosis, coronary heart disease and high blood pressure [31]. Oleic acid, a monounsaturated fatty acid (MUFA), is the second-most abundant oil in oak silkworm pupal oil (30.97%). The main saturated acid in the oak silkworm pupal oil is palmitic acid, followed by stearic acid.

## 3. Experimental Section

### 3.1. Materials

Oak silkworm pupae were obtained from Shenyang Agricultural University (Shenyang, China). The fresh mulberry silkworm pupae were supplied by Anhui Sericultural Institution (Hefei, China). Samples were vacuum dried at 60 °C to a stable moisture content of less than 5%, and then stored in airtight plastic bags at 4 °C. The samples were ground in a blender before extraction.

### 3.2. Reagents

Carbon dioxide (99.99% purity), in cylinders, was purchased from Jinwang Gas Co. (Anhui, China). HPLC grade hexane for GC and GC/MS were purchased from Beijing Chemical Co. (Beijing, China). Other solvents and chemicals were obtained commercially and were of analytical grade.

### 3.3. Oil Determination by Soxhlet

Total oil content of the oak silkworm pupae was determined by Soxhlet. The milled samples were extracted by Soxhlet apparatus for 6 h at 65 °C using petroleum ether (boiling range 30–60 °C) as the solvent, then evaporated to obtain pupal oil, according to the ISO 659 standard method. The oil yield obtained by solvent extraction was 28.08 ± 0.45/100 g of dry pupae.

### 3.4. Oil Extraction by Supercritical Carbon Dioxide

All experiments were performed using a Supercritical Fluid Extractor (SFE) system, provided by Nantong Hua’an Co., Ltd. (Model HA220-50-06, Jiangsu, China). The procedure of oil extraction by supercritical carbon dioxide followed the descriptions by Wei *et al*. [6]. The apparatus includes a chiller filled with a mixture of water and ethanol, a high-pressure pump, an extraction vessel (35 cm height, 6 cm inside diameter, and 1 L capacity), separators (named separator I and separator II, respectively), heating chambers, valves and a flow meter. Carbon dioxide from a cylinder was passed through a chiller kept at 2 °C and pumped into the extractor using a high-pressure pump. The pressure in the extractor was controlled to the desired value by adjusting the pressure regulating valve. When both the pressure and temperature reached the required levels, which were controlled to an accuracy of ±0.02 MPa and ±0.5 °C, respectively, the extraction began. The flow rate of carbon dioxide was measured with a flow meter and regulated by the frequency of the pumping stroke. After each extraction, the oil was collected in the first separator while volatile components were recovered in the second separator. The amount of extracted oil was determined gravimetrically after collection, and then the extraction yield shown as the ratio of the mass of extracted oil to the mass of silkworm pupae loaded in the extraction vessel, as Equation (2):

(2) Oak silkworm oil yield=mass of extracted oil/mass of dried material×100%

### 3.5. Chromatographic Procedure

To determine the fatty acid composition of oak silkworm pupal oil, the oils were firstly converted into fatty acid methyl esters via esterification reaction, and then analyzed by GC and GC-MS. The pupal oils (300 mg) were initially dissolved with 3 mL 1 mol/L KOH methanol solution. The vials with reaction mixtures were sealed and heated in a water bath at 60 °C for 30 min (until oil droplets completely disappeared). After cooling, the mixture was etherified with 40 mL 12.5% H_2_SO_4_ methanol solution. After shaking under 38 °C, 130 r/min for 12 h, the organic layer was extracted by hexane and washed several times with saturated NaCl solution. Finally, it was dried over anhydrous Na_2_SO_4_, centrifuged and filtered, and then the organic layer with the final volume of 1 mL was injected into the GC system. Methyl esters of carboxylic acids were detected by GC/MS and quantified by GC/FID [32,33].

GC/MS analysis was run on a QP2010 gas chromatography/mass spectrometry instrument. For oak silkworm pupal oil analysis, the GC was fitted with a capillary DB-WAX column (0.25 μm film thickness, 30 m length and inner diameter 0.25 mm). The operating conditions were as follows: injector temperature 250 °C; split ratio, 50:1; detector temperature, 260 °C. The temperature program was: from 200 to 230 °C (3 °C/min). The mass spectrometry (MS) conditions were as follows: ionization voltage, 70 V; ion source temperature, 260 °C; scan range, *m/z* 35–500. 1 μL of oak silkworm pupal oil sample was injected into the system. The identification of the components was carried out, based on computer matching with Adams and the National Institute of Standards and Technology (NIST)/Environmental Protection Agency (EPA)/National Institutes of Health (NIH) version 2.0 d mass spectral libraries. The components of extracts were also identified by comparing their retention times to those in the Adams library. The percentage composition was computed by the normalization method from the GC (FID) peak areas [6].

### 3.6. Experimental Design for Response Surface Methodology

In this study, response surface methodology (RSM) and central composite design were used for determining the optimal extraction pressure (MPa, X1), temperature (°C, X2), time (min, X3), and CO_2_ flow rate (mL/min, X4) of SFE process to obtain a high extraction yield. These independent variables and their levels (Table 1) were selected, based on the preliminary experiments in our laboratory (data not shown). The experimental design was based on a central composite rotatable design (CCRD) consisting of four variables, including: 31 experimental settings with 16 (2^4^) factorial points, 8 star points (star distance is 0), and 7 central points as shown in Table 2. The oil recovery (Y) at each design point was recorded. Triplicate extractions were carried out at all the design points. A second-order polynomial equation was used to express the oil yield (Y) as a function of the independent variables. The experiments were run in random order to minimize the effects of unexpected variability in the observed responses due to extraneous factors. The experimental design included 31 experiments of five variables at five levels (−2, −1, 0, +1, +2). Table 1 gives the range of variables employed. The actual set of experiments performed (experimental runs 1–31) and the yield of the oak silkworm pupal oil are shown in Table 2.

A second-order polynomial equation was developed to study the effects of variables on the yield. The equation indicates the effect of variables in terms of linear, quadratic and cross-product terms: The second-order polynomial fitted [34] was (Equation (3)):

(3) $$\text{Y}=\beta_{0}+\sum_{i=1}^{4}\beta_{i}X_{i}+\sum_{i=1}^{4}\beta_{ii}X_{i}^{2}+\sum \sum_{i<j=1}^{4}\beta_{ij}X_{i}X_{j}$$

where Y is the oak silkworm pupal oil (%), X_i_ and X_j_ are the levels of variables (extraction pressure, temperature, time, and CO_2_ flow rate), β_0_ the constant term, β_i_ the coefficient of the linear terms, β_ii_ the coefficient of the quadratic terms and β_ij_ the coefficient of the cross-product terms. The experimental plan was designed and the results obtained were analyzed using SAS 9.0 software [35] to build and evaluate models and to plot the three-dimensional response surface curves.

## 4. Conclusions

In the process of extraction of oak silkworm pupal oil with supercritical carbon dioxide, the response surface methodology (RSM) was applied to achieve an ideal condition. With SC-CO_2_, the yield of the oak silkworm pupal oil yield was found to be dependent on the linear term of extraction pressure, time, and the quadratics of pressure, temperature, and extraction time as well as the interactions between extract pressure and temperature, and temperature and CO_2_ flow rate. A polynomial regression model was established to describe the experimental results. The optimal condition for oak silkworm pupal oil yield was at 28.03 MPa, 35.31 °C, 1.83 h and 20.26 L/h. In this condition, the predicted proportion of oil yield reached 26.18%. The oak silkworm pupal oil contains eight fatty acids, and is rich in unsaturated fatty acids which accounting for 77.29% of the total.

## Figures and Tables

**Table 1:** Uncoded and coded levels of independent variables used in the RSM design.

Independent Variables	Symbols	Variable Levels				

		−2	−1	0	1	2
Pressure (MPa)	X1	15	20	25	30	35
Temperature (°C)	X2	25	30	35	40	45
Extraction time (h)	X3	0.5	1.0	1.5	2.0	2.5
CO_2_ flow rate (L/h)	X4	11	16	21	26	31

**Table 2:** Central composite rotatable design for the optimization of Supercritical Fluid Extractor (SFE) oak silkworm pupal oil and the values of observed responses.

	Coded Variable				Process Variable				
		
Design Point	X1	X2	X3	X4	Pressure (MPa)	Temperature (°C)	Time (h)	CO_2_ Flow Rate (L/h)	Oil Yield (Y, %)
1	−1	−1	−1	−1	20	30	1.0	16	22.38 ± 0.20
2	−1	−1	−1	1	20	30	1.0	26	24.34 ± 0.33
3	−1	−1	1	−1	20	30	2.0	16	23.73 ± 0.18
4	−1	−1	1	1	20	30	2.0	26	24.25 ± 0.45
5	−1	1	−1	−1	20	40	1.0	16	24.19 ± 0.26
6	−1	1	−1	1	20	40	1.0	26	24.00 ± 0.34
7	−1	1	1	−1	20	40	2.0	16	24.75 ± 0.15
8	−1	1	1	1	20	40	2.0	26	24.62 ± 0.38
9	1	−1	−1	−1	30	30	1.0	16	24.65 ± 0.22
10	1	−1	−1	1	30	30	1.0	26	24.98 ± 0.51
11	1	−1	1	−1	30	30	2.0	16	25.09 ± 0.16
12	1	−1	1	1	30	30	2.0	26	26.04 ± 0.32
13	1	1	−1	−1	30	40	1.0	16	24.54 ± 0.21
14	1	1	−1	1	30	40	1.0	26	23.33 ± 0.43
15	1	1	1	−1	30	40	2.0	16	25.87 ± 0.18
16	1	1	1	1	30	40	2.0	26	24.67 ± 0.15
17	−2	0	0	0	15	35	1.5	21	23.01 ± 0.20
18	2	0	0	0	35	35	1.5	21	24.98 ± 0.32
19	0	−2	0	0	25	25	1.5	21	22.68 ± 0.66
20	0	2	0	0	25	45	1.5	21	24.37 ± 0.19
21	0	0	−2	0	25	35	0.5	21	23.36 ± 0.48
22	0	0	2	0	25	35	2.5	21	25.09 ± 0.13
23	0	0	0	−2	25	35	1.5	11	24.58 ± 0.27
24	0	0	0	2	25	35	1.5	31	25.68 ± 0.37
25	0	0	0	0	25	35	1.5	21	26.00 ± 0.10
26	0	0	0	0	25	35	1.5	21	25.98 ± 0.17
27	0	0	0	0	25	35	1.5	21	25.19 ± 0.15
28	0	0	0	0	25	35	1.5	21	26.09 ± 0.09
29	0	0	0	0	25	35	1.5	21	26.01 ± 0.20
30	0	0	0	0	25	35	1.5	21	26.18 ± 0.12
31	0	0	0	0	25	35	1.5	21	25.95 ± 0.17

**Table 3:** ANOVA table for the fitted quadratic polynomial model of SFE extraction conditions.

Source	Degrees of Freedom	Sum of Square	Mean Square	*F*-value	Probability (p)
Model	14	30.9368	2.2098	12.7669	0.0001
Lack of fit	10	2.1212	0.2121	1.9636	0.2112
Pure error	6	0.6482	0.1080		
Cor total	30	33.7062			

*R*^2^ = 0.9178; *Adj. R*^2^ = 0.8459.

**Table 4:** Regression coefficient of polynomial function of response surface of oil yield.

Source	Degrees of Freedom	Sum of Square	Mean Square	F-value	Probability (p)
X_1_	1	4.9051	4.9051	28.3391	0.0001
X_2_	1	0.6305	0.6305	3.6427	0.07442
X_3_	1	4.2252	4.2252	24.4110	0.000148
X_4_	1	0.4347	0.4347	2.51149	0.1326
X_1_ × X_1_	1	5.5081	5.5081	31.8228	0.0001
X_1_ × X_2_	1	1.6965	1.6965	9.8015	0.006451
X_1_ × X_3_	1	0.1871	0.1871	1.0807	0.3140
X_1_ × X_4_	1	0.6765	0.6765	3.9085	0.06554
X_2_ × X_2_	1	8.8522	8.8522	51.1432	0.0001
X_2_ × X_3_	1	0.07426	0.07426	0.4290	0.5218
X_2_ × X_4_	1	2.6325	2.6325	15.2092	0.001274
X_3_ × X_3_	1	4.1594	4.1594	24.0306	0.00016
X_3_ × X_4_	1	0.03516	0.03516	0.2031	0.6583
X_4_ × X_4_	1	0.6882	0.6882	3.9761	0.06348

**Table 5:** Fatty acid composition of two kinds of silkworm pupae oil.

Composition	Relative Content/%	

	Oak Silkworm Pupal Oil	Mulberry Silkworm Pupal Oil
Palmitic acid	19.92	22.77
Palmitoleic acid	4.77	0.60
Heptadecanoic acid	0.60	ND
Stearic acid	1.99	6.69
Oleic acid	30.97	26.01
Linoleic acid	6.89	5.90
α-linolenic acid	34.27	38.02
0(*z*),13(*z*),16(*z*)-nonadecatrienoic acid	0.39	ND

ND: Not detected.
